# Close Contact Transillumination Light Guides Surgeon to Vaginal Point Aa: Pharus Method for Robot‐Assisted Sacrocolpopexy

**DOI:** 10.1111/ases.13412

**Published:** 2024-11-23

**Authors:** Akiko Yoshida Ueno, Takayuki Sato, Michiya Kobayashi, Shinya Wakatsuki, Takaomi Namba, Kazutoshi Hayashi

**Affiliations:** ^1^ Department of Obstetrics and Gynecology Kochi Health Sciences Center Kochi Japan; ^2^ Graduate School of Integrated Arts and Sciences, Medicine Program (Doctoral Course) Kochi Medical School Nankoku Japan; ^3^ Department of Cardiovascular Control Kochi Medical School Nankoku Japan; ^4^ Department of Human Health and Medical Sciences, Hospital Administration Section Kochi Medical School Nankoku Japan

**Keywords:** endoscopic tattooing, firefly mode, intraoperative navigation, robot‐assisted sarcocolpopexy, vaginal transillumination method

## Abstract

**Introduction:**

In robot‐assisted sacrocolpopexy (RSC) for patients with cystocele, accurate identification of the vaginal point Aa from the serosal side is crucial for surgical mesh placement in the appropriate position. We developed a novel Pharus method for exactly locating the point Aa for RSC.

**Methods:**

In the Pharus method, the tip of a rigid endoscope was placed directly on the vaginal point Aa. In a preliminary experiment, we observed LED lights with different wavelengths of 450–870 nm using the Firefly imaging system to evaluate which wavelengths of light were captured by the Firefly mode. In a clinical study, the Pharus method was employed in four patients with Stage II or more advanced cystocele undergoing RSC. For comparison, a near‐infrared fluorescence method by indocyanine green (ICG) tattooing at the point Aa was also performed. The visibility of each method was evaluated under Firefly‐mode imaging.

**Results:**

In the preliminary experiment, visible LED lights with wavelengths ≤ 720 nm, and near‐infrared LED lights with wavelengths ≥ 830 nm were detected by the Firefly mode. In RSC using the Pharus method, the point Aa of each patient was clearly highlighted as a green spot from the serosal side by the endoscopic white light penetrating the vaginal wall with a thickness of 3.3–4.6 mm. Compared with the ICG tattooing method, the Pharus method showed superior visibility in all patients.

**Conclusion:**

The transillumination light effectively guided the surgeon to the vaginal point Aa, which can be likened to the Latin word “pharus,” meaning lighthouse.

## 1 Introduction

Robot‐assisted sacrocolpopexy (RSC) has been widely adopted due to its benefits, including 3D visualization, tremor reduction, and the ability to suture with multi‐jointed instruments. Accurate identification of the vaginal point Aa is crucial for patients with cystocele, as it determines the site of mesh fixation and whether the cystocele will be corrected. In the Pelvic Organ Prolapse Quantification System, the vaginal point Aa is defined as a midline point on the anterior vaginal wall, 3 cm proximal to the external urethral meatus, and corresponds to the urethrovesical junction [1]. The precise identification and correction of the vaginal point Aa is essential for improving symptoms in women with incontinence due to urethral hypermobility. There is also a risk of bladder injury if the vesicovaginal space is incorrectly dissected. In fact, the procedural complications of RSC are reported to be 2.7%, with nearly half (48.6%) of these being bladder injuries [2]. Currently, there is no established method for identifying the targeted vaginal point Aa from the serosal side during RSC procedures.

In the present study, we developed a novel method for the precise determination of the vaginal point Aa with a close contact transillumination light of a rigid endoscope. This method has been designated the “Pharus” method, deriving its name from the Latin word for lighthouse. Initially, a preliminary experiment was conducted to clarify the response of the Firefly imaging system of the da Vinci Xi surgical system to light of different wavelengths. Subsequently, both Pharus method and near‐infrared fluorescence method by indocyanine‐green (ICG) tattooing were evaluated to locate the vaginal point Aa during RSC procedures.

## 2 Materials and Methods

### 2.1 Ethical Considerations

Written informed consent was obtained from each patient for intraoperative use of new methods and academic reporting, and research approval was granted by the Clinical Research Review Board of Kochi Health Sciences Center (Approval Nos. 243008 and 241 036). The study was performed under the Declaration of Helsinki.

### 2.2 Preliminary Experiment

The objective of this protocol was to ascertain which wavelengths of light are rendered green in the Firefly imaging system of the da Vinci Xi surgical system (Intuitive Surgical, California, USA). The experiment was conducted on the assumption that a 5‐mm 0° rigid endoscope (Olympus, WA50372B, Tokyo, Japan) and a white light‐emitting diode (LED) light source with a wavelength range of 380–720 nm (Olympus CLL‐S700) were being utilized as contact transillumination at the vaginal point Aa. An array of LED lights with visible wavelengths of 450, 535, 590, 625, and 720 nm and near‐infrared wavelengths of 750, 780, 830, 850, and 870 nm was photographed by the normal and sensitive Firefly modes of the Firefly imaging system [3]. In the normal mode, images were captured with the internal light source operating in white light illumination. In the sensitive Firefly mode, only the light emitted from the LED array could be captured, as there were neither fluorescent materials nor internal white light illumination within the field of vision.

### 2.3 Clinical Study

To ensure the safety of the Pharus method, the temperature of the rigid endoscope tip was measured with a thermocouple thermometer. It was found that the temperature remained below 30°C when the output level of the white LED light source was set to 2/17 of maximum. Consequently, the fixed level of the white LED light source was used throughout the clinical study.

Four patients with Stage II or more advanced cystocele undergoing RSC were enrolled in the present study. Before initiating the RSC procedures, the attending surgeon and an assistant surgeon marked the mucosa of the vaginal point Aa with a pen. Following a supracervical hysterectomy and bilateral salpingo‐oophorectomy, a single anterior repair in RSC procedures was performed with a piece of polytetrafluoroethylene mesh measuring 3 by 16 cm. The visibility of the vaginal point Aa from the serosal side by the Firefly imaging system was evaluated during RSC procedures using the Pharus and ICG tattooing methods.

In accordance with the Pharus method, the assistant surgeon positioned the rigid endoscope in close contact with the designated point on the mucosa, as directed by the attending surgeon at the console. The images of the normal and Firefly modes were recorded for further analysis of visibility.

For the ICG tattooing method, a diluted solution of ICG (Diagnogreen; Daiichi Sankyo, Tokyo, Japan) with a concentration of 0.025–2.5 mg/mL and a volume of 0.1–0.5 mL was injected submucosally at the vaginal point Aa. The images of the normal and Firefly modes were recorded for further analysis of visibility. The Pharus method was performed initially, followed by the ICG tattooing method.

In the postoperative analysis of images obtained during RSC procedures, the visibility of the vaginal point Aa was evaluated on Price's Visibility Scale by the surgeons, irrespective of the attending surgeon's assessment: 0, not seen; 1, seen with difficulty; 2, seen easily [4].

## 3 Results

### 3.1 Preliminary Experiment

**FIGURE 1:**
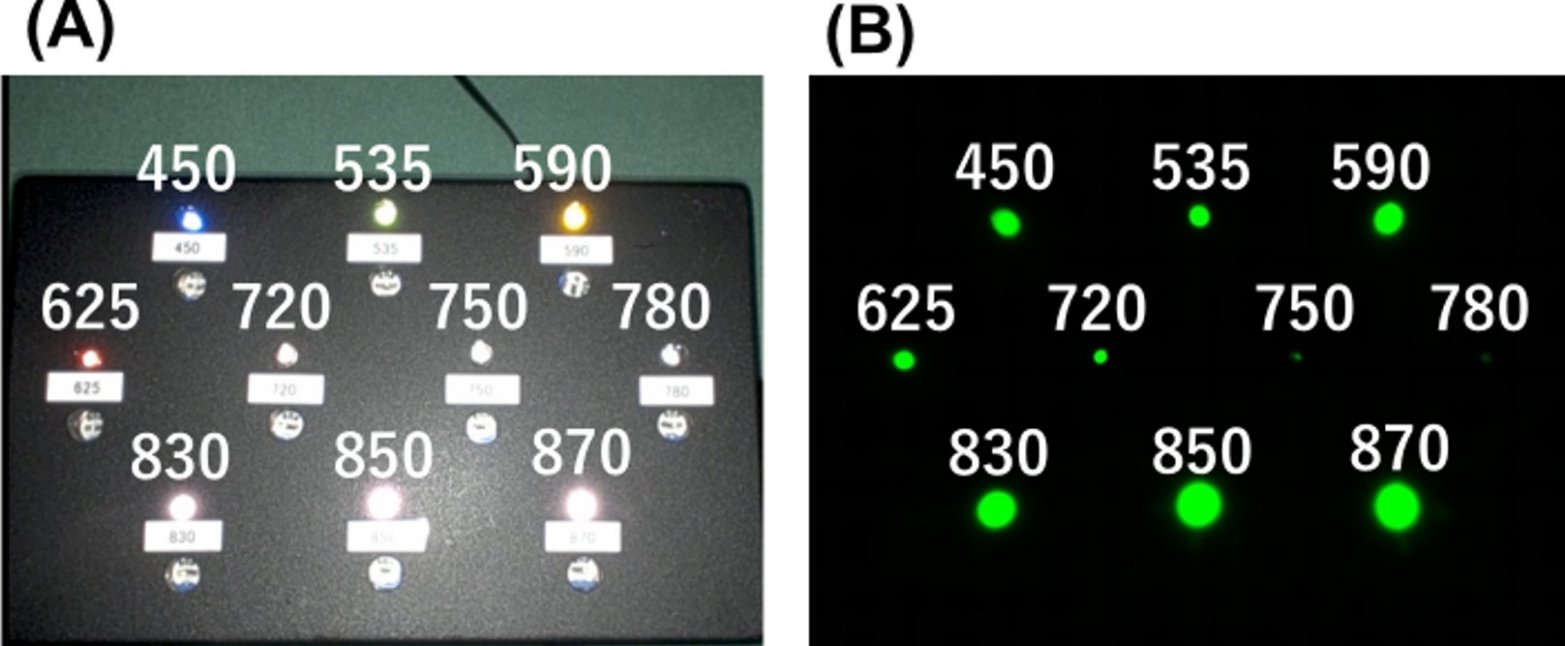
Images of the normal (A) and the sensitive Firefly modes (B) of an array of LED lights with varying wavelengths of 450–870 nm. The values indicate the wavelengths of the LED lights. In the normal‐mode image, each visible LED light with a wavelength of 450–720 nm appeared in its proper color. In the sensitive Firefly‐mode image, both visible LED lights and near‐infrared LED lights with wavelengths of 830–870 nm appeared in green.

In the normal‐mode images, visible LED lights of wavelengths from 450 to 720 nm showed reasonable chromaticity, respectively (Figure 1A). The dark background of the sensitive Firefly mode made it evident that both visible LED lights with wavelengths of ≤ 720 nm and near‐infrared LED lights with wavelengths of ≥ 830 nm were rendered in green (Figure 1B). Conversely, LED lights with wavelengths of 750 and 780 nm were not captured by the sensitive Firefly mode.

### 3.2 Clinical Study

**FIGURE 2:**
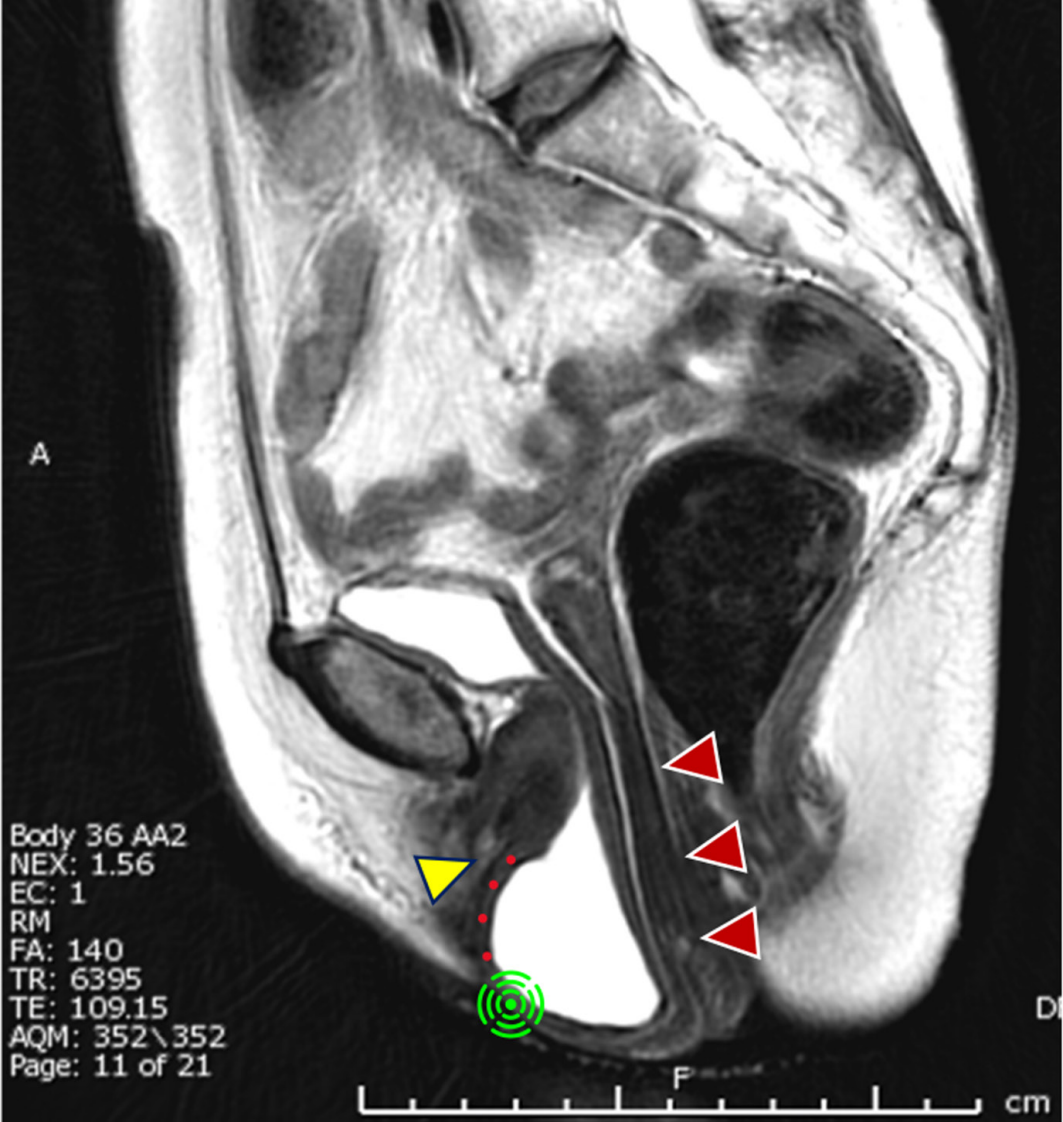
MRI sagittal view. The red arrows indicate uterine cervix and cervical elongation. The anterior vaginal wall, in conjunction with the bladder filled with urine, had undergone a severe prolapse, resulting in a Stage 3 cystocele. The yellow arrow indicates the external urethral meatus. The vaginal point Aa, indicated by a green symbol, was situated 3 cm proximal to the external urethral meatus.

The Pharus or ICG method was observed to have no adverse effects on any of the patients. The clinical characteristics of the patients are presented in Table 1. The thickness of the vaginal wall at the point Aa was estimated to be between 3.3 and 4.6 mm based on the preoperative MRI scans (Table 2). A sagittal MRI of a typical Stage III patient (Case 4) is shown in Figure 2. In the patient who exhibited severe vaginal prolapse and cervical elongation, as illustrated here, dissection of the vesicovaginal space was anticipated to be a challenging procedure.

**TABLE 1:** Characteristics of four patients.

Characteristics	Value
Age, years	65 (54–77)
Body mass index, kg/m^2^	22.5 (17.8–26.0)
Parity	2.5 (2–4)
POP‐Q Score	
Point Ba, cm	+2 (+1 to +3)
Point C, cm	0.5 (−2 to +5)
Point Bp, cm	−2 (−1 to −3)
POP‐Q Stage	
II	1
III	3
IV	0

*Note:* Values are expressed as median (range) or *n*.

Abbreviations: Point Ba, POP‐Q Score for the anterior vaginal wall; Point Bp, POP‐Q Score for the posterior vaginal wall; Point C, POP‐Q Score for the uterine cervix; POP‐Q, Pelvic Organ Prolapse Quantification.

**TABLE 2:** Comparison of visibility of the vaginal point Aa.

Case no	VWT, mm	Visibility scale		ICG dose	
		Pharus	ICG	Conc, mg/mL	Vol, mL
1	3.6	2	0	0.025	0.1
2	4.6	2	0	0.25	0.1
3	3.3	2	2	2.5	0.5
4	3.8	2	0	2.5	0.5

Abbreviations: Conc, concentration; ICG, indocyanine green; Vol, volume; VWT, vaginal wall thickness.

**FIGURE 3:**
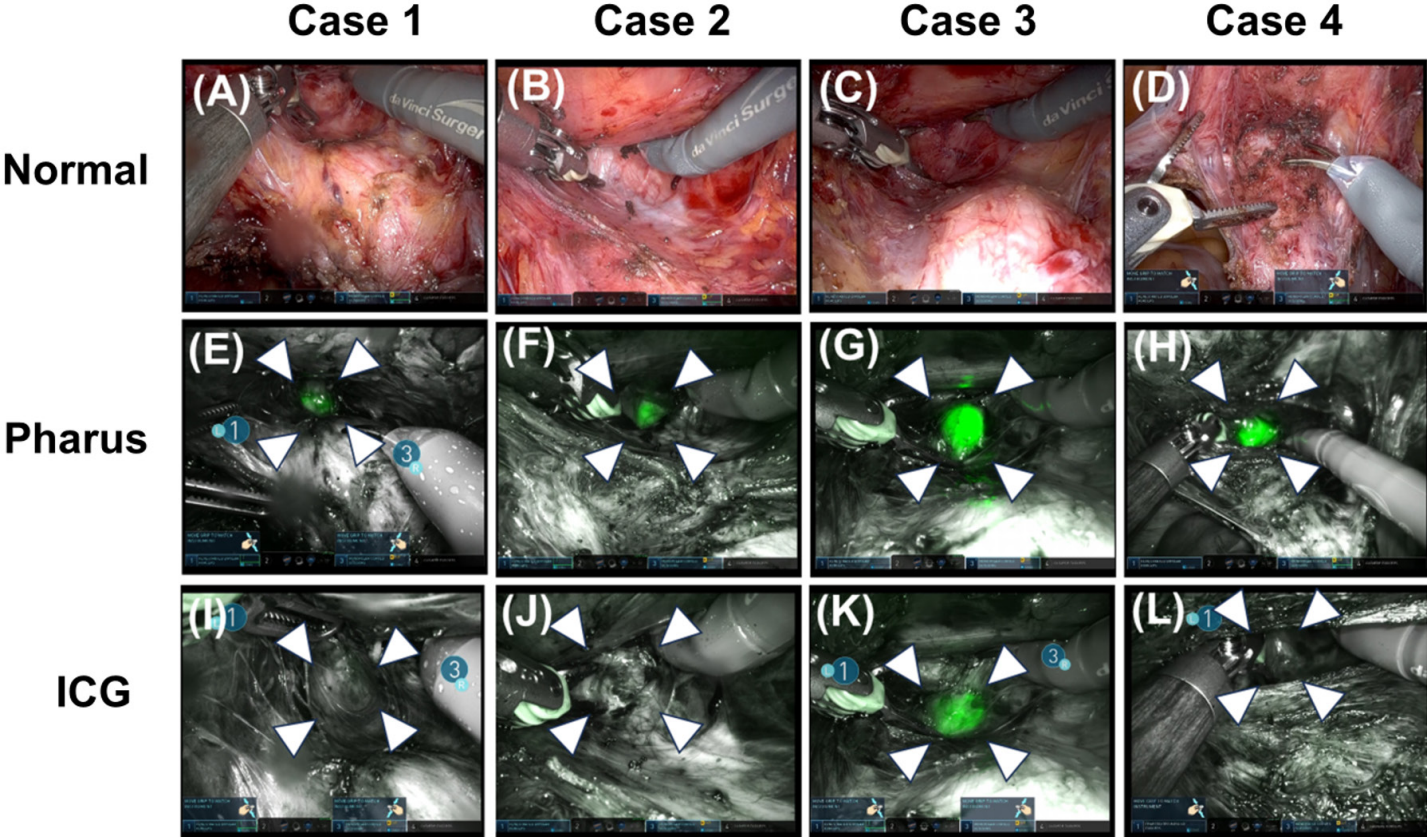
Normal‐ and Firefly‐mode images of the operative field during dissection of the vesicovaginal space in RSC procedures. The areas enclosed by white arrows indicate the vaginal point Aa. In the top row (A)–(D) show the normal‐mode images for Cases 1–4, respectively. In the middle row (E)–(H) show the Firefly‐mode images of the Pharus method for Cases 1–4, respectively. The bottom row (I)–(L) display the Firefly‐mode images of the ICG tattooing method for Cases 1–4, respectively. See text for detailed explanation.

Normal‐ and Firefly‐mode images of the operative field during dissection of the vesicovaginal space are shown in Figure 3. As shown in the top row of images (A)–(D), the position of vaginal point Aa was not identifiable on the normal‐mode images of the serosal aspect in any patient. In contrast, the Pharus method clearly and accurately guided the vaginal point Aa of each patient, as illustrated in the middle row of Firefly‐mode images (E)–(H). Except for Case 3, the ICG fluorescence method with its tattooing on the vaginal mucosa failed to reveal the vaginal point Aa on the serosal side, as shown in the bottom row of the Firefly‐mode images (I)–(L).

The evaluation of the visibility of the vaginal point Aa for each patient using the Pharus and ICG tattooing methods is summarized in Table 2. Compared with the ICG tattooing method, the Pharus method showed superior visibility in all patients.

The intraoperative video of Case 4 is demonstrated in Video S1. Upon initiating of the vesicovaginal dissection, the Pharus method revealed the vaginal point Aa on the serosal aspect behind the 3.8‐mm thick vaginal wall. This enabled the surgeon to perform a safe dissection of the vesiovaginal space without bladder injury and to accurately aim at the vaginal target during RSC procedures.

## 4 Discussion

### 4.1 Specific Consideration for Pharus Method

In a case report published in abstract form by Walker et al. [5], it was demonstrated that the white light emitted from the tip of the hysteroscope could penetrate the uterine isthmocele, which had a thin wall thickness of 1.4 mm. Furthermore, the Firefly mode was shown to be effective in delineating the extent of the isthmocele by transillumination. While their findings prompted us to consider the potential of using the white light emitted from the tip of a rigid endoscope to identify the vaginal point Aa during RSC procedures, two technical issues emerged.

First, it is necessary to clarify the specifications of the endoscope illumination light required to be captured through the wall of the uterine and vagina by the Firefly mode. This mode has been designed to visualize near‐infrared light or ICG fluorescence as green in the grayscale image. While the specifications of the white light source employed by Walker et al. were not documented, it is not implausible to hypothesize that it was a xenon lamp, which has a broad band wavelength ranging from visible to near‐infrared light. If this was indeed the case, the success in delineating the extent of the isthmocele by transillumination may have been attributed to the inclusion of near‐infrared light in the xenon lamp, as opposed to visible light. It is therefore essential to clarify this technical issue to confirm the applicability of an endoscope white LED light, which lacks near‐infrared illumination, as a transillumination source for the Pharus method. The rationale behind the selection of an LED light source for the Pharus method was its distinctive capacity to emit minimal heat, thereby reducing the risk of tissue burn injury. In some of the most widely commercially available endoscope systems utilizing xenon lamps, the temperature of the tip has been reported to exceed 60°C at maximum [6].

The preliminary experiment yielded the following results: the sensitive Firefly mode was observed to capture visible LED lights with wavelengths of blue (450 nm), green (535 nm), yellow (590 nm), red (625 nm), and far red (720 nm) as well as near‐infrared LED lights (830, 850, and 870 nm) with a wavelength of ICG fluorescence. To capture ICG fluorescence, it would be necessary to incorporate an optical rejection filter into the front of an image sensor of the Firefly imaging system, with the aim of eliminating a wavelength band of laser light for ICG excitation (760–800 nm). This specification for a requirement is consistent with the observation that LED lights with wavelengths of 750 and 780 nm were not captured by the sensitive Firefly mode. In conclusion, the results of the preliminary experiment indicate that a specific white light, such as a xenon lamp, is not a prerequisite for the Pharus method. Furthermore, it is essential to recognize that the green hue observed in the Firefly mode does not necessarily indicate the presence of near‐infrared light or near‐infrared fluorescence.

Second, the presence of a thicker vaginal wall over 2.5 mm, as shown in an earlier study by Bray et al. [7], made it challenging to identify the exact location of the vaginal point Aa by transmural penetration of the widely dispersed light that was broadly projected onto the vaginal mucosa. In the Pharus method, therefore, the tip of a rigid endoscope with a white LED light source, which has low heat output, was placed directly on the vaginal point Aa, so that effective transillumination through the vaginal wall was expected to be clearly highlighted as a green spot in the grayscale image by the Firefly mode. To the best of our knowledge, this is the first study to demonstrate the usefulness of the close contact transillumination method for locating the vaginal point Aa from the serosal surface in RSC.

### 4.2 Impact of Pharus Method on RSC

As demonstrated by the present result of the visibility, the Pharus method enabled the surgeon to accurately identify the vaginal point Aa in RSC in all patients. In addition to the advantages of not requiring drug injection, invasive procedure, or special technique, the Pharus method had a notable strength of clearly revealing the vaginal point Aa on the serosal surface behind the 4.6‐mm thick vaginal wall.

Before the implementation of the Pharus method, an attending surgeon operating the da Vinci Xi surgical system at the console, who was identifying the vaginal point Aa from the serosal surface, directed an assistant surgeon to repeatedly push the point Aa on the vaginal mucosa with his or her fingers. However, in practice, it was often challenging for the attending surgeon to accurately identify the vaginal point Aa from the serosal side. In such a case, the attending surgeon was obliged to withdraw from the console and relocate to the patient's groin side following the placement of the robotic surgical forceps tip in close contact with the serosal surface of the presumed vaginal point Aa. Subsequently, the attending surgeon was required to confirm the correct positioning of the forceps tip at the vaginal point Aa through palpation of the vaginal mucosa. Therefore, a new operative technique that eliminates the need for these time‐consuming procedures for RSC has been anticipated. Conversely, the Pharus method has enabled the attending surgeon at the console to ascertain the vaginal point Aa with immediate precision. Moreover, this method would be also applicable to other surgical procedures. For example, it could be used as a landmark technique for the edge of the vaginal cuff [8, 9].

### 4.3 Comparison of Pharus Method With ICG Tattooing Method

The ICG tattooing method of the vaginal wall has been investigated in cases of deep endometriosis and rectal cancer invasion, with a concentration of 2.5 mg/mL and an injection volume of 0.5–1.0 mL. However, there is no documentation of the optimal dosage of ICG injection for its tattooing at the vaginal point Aa. Despite the utilization of various doses in the present study to visualize the vaginal point Aa, the visibility did not exceed that achieved through the Pharus method. However, due to the limited number of patients included in this study, it is not possible to draw any definitive conclusions.

In addition to the inherent risks associated with the low visibility and unstable reproducibility of the ICG tattooing method, this method has several inherent limitations. The administration of ICG into the vaginal wall has not been approved by the pharmaceutical regulatory authorities in Japan or other countries. Therefore, the unintentional use of ICG necessitates the approval of institutional review boards, which seems clinically impractical. While anaphylactic shock is an uncommon occurrence, with a frequency of 1 in 40 000 [10], there is an inherent risk associated with the ICG tattooing method. Therefore, the Pharus method is considered to be more practical and safer.

### 4.4 Technical Limitations

Although the usefulness of the Pharus method for locating the vaginal point Aa was evaluated with the Firefly imaging system, other imaging systems are commercially available. Therefore, differences in the visibility of the vaginal point Aa using the Pharus method between models of imaging systems should be investigated.

In the present study, a LED light source was selected for use with the Pharus method due to its low heat output. It is recommended that the temperature of a rigid endoscopic tip be monitored before use when employing the Pharus method with a xenon light source with high heat output [6].

## 5 Conclusion

The Pharus method was developed using a close contact transillumination light of a rigid endoscope to locate the vaginal point Aa in RSC. The Pharus method demonstrated remarkable efficacy in clearly revealing the vaginal point Aa on the serosal surface behind the 4.6‐mm thick vaginal wall, without the need for drug injection, invasive procedure, or special technique. The Pharus method is a valuable tool for accurately identifying the vaginal point Aa in RSC.

## Author Contributions

The manuscript has been read and approved by all the named authors, and all those who satisfy the criteria for authorship are listed.

## Ethics Statement

This study was approved by the Research Ethics Committee of our hospital. The patient's identity was protected.

## Consent

Written informed consent was obtained from all patients for publication of the accompanying images.

## Conflicts of Interest

The authors declare no conflicts of interest.

## Supporting information


**Video S1.** This video presents surgical procedures of the dissection of the vesicovaginal space in RSC procedures. A 68‐year‐old woman (Case 4 in Table 2) had a history of two vaginal deliveries and no abdominal surgery. Following the onset of menopause at the age of 50, the patient had been experiencing symptoms of pelvic organ prolapse for a period exceeding 10 years. The uterine body had already been removed, and the cervix was retracted cranially with the sutures that had been used for closure of the cervix in a previous supracervical hysterectomy. The peritoneum around the bladder was lifted ventrally by pulling a suture on each side. Both cystocele and uterine prolapse were severe (see Figure 2), accompanied by cervical elongation, which made identification of the vesicovaginal space challenging. The proximity to the bladder and tendency for easy bleeding were evident. Upon initiating the dissection, the Pharus method revealed the vaginal point Aa on the serosal aspect behind the 3.8‐mm thick vaginal wall. This prompted the surgeon to recognize that the precise location of the vaginal point Aa was to the left of the initial assumption, allowing for a correction of the dissection plane. Subsequently, an ICG injection with a concentration of 2.5 mg/mL and a volume of 0.5 mL was made at the vaginal point Aa, but ICG fluorescence was not observed at all.

## Data Availability

The data that support the findings of this study are available on request from the corresponding author. The data are not publicly available due to privacy or ethical restrictions.
